# A modification to heptad repeat 1 of gp41 improves yield and/or quality of soluble pre-fusion HIV-1 envelope glycoprotein trimers

**DOI:** 10.1128/jvi.00913-25

**Published:** 2025-08-27

**Authors:** Devidas N. Chaturbhuj, Kwinten Sliepen, Albert Cupo, Benjamin Steinberg, Simon Kazimierczyk, Tarek Munawar, Kyle Kramer, Anila Yasmeen, Thales G. Andrade, Wen-Hsin Lee, Lara van der Maas, Grace Gibson, Oscar Feliciano, Ivan del Moral Sanchez, Edith Schermer, Rhianna Bronson, Alison Benner, Madhu Prabhakaran, Rosemarie Mason, P. J. Klasse, Andrew B. Ward, Gabriel Ozorowski, Rogier W. Sanders, John P. Moore

**Affiliations:** 1 Department of Microbiology and Immunology, Weill Cornell Medicine 12295, New York, New York, USA; 2 Department of Medical Microbiology, Amsterdam University Medical Centres 522567, Amsterdam, the Netherlands; 3 Department of Integrative Structural and Computational Biology, The Scripps Research Institute 579993, La Jolla, California, USA; 4 Vaccine Research Center, National Institute of Allergy and Infectious Diseases, National Institutes of Health 35037, Bethesda, Maryland, USA; University Hospital Tübingen, Tübingen, Germany

**Keywords:** HIV-1, SOSIP, trimer, gp41, heptad repeat, Env glycoprotein, vaccine, global virus panel

## Abstract

Native-like HIV-1 envelope glycoprotein (Env) trimers, exemplified by the SOSIP design, are widely used as immunogens, analytical antigens, and for structural studies. These vaccine research and development programs require trimers that are based on multiple HIV-1 genotypes. While a wide range of protein engineering strategies can produce SOSIP trimers from most Env gene sequences, there are still examples of trimers that are expressed only at impractically low yields or that are unstable. Accordingly, additional protein modifications aimed at overcoming such limitations need to be evaluated. Here, we describe a new heptad repeat 1 modification of gp41, known as dPG, that helps to further stabilize the gp41 component of prototypic and germline-targeting SOSIP trimers in the pre-fusion state and thereby increases post-purification yields substantially. The dPG modification involves a deletion (d) at the highly conserved 566 position that disrupts the heptad repeat and introduces proline (P) and glycine (G) substitutions at positions 567 and 568, respectively. We show that the dPG strategy reinforces previously described stabilization changes in existing SOSIP trimers and can rescue otherwise problematic trimer constructs. The latter includes trimers used to target or analyze human germline antibodies and others derived from the global HIV-1 neutralization panel. In summary, the dPG modification strategy can increase the yield and/or quality of Env trimers that are otherwise difficult to produce.

**IMPORTANCE:**

Stabilized, soluble, pre-fusion SOSIP trimers are widely used in HIV-1 Env vaccine research. Protein engineering techniques have identified multiple ways to stabilize SOSIP trimers from a range of genotypes. However, some SOSIP trimers remain difficult to express at adequate yields and/or purity, so there is a need for additional modifications. Here, we identified a sequence change, designated dPG, to the gp41 subunit that increases the yield and/or quality of various otherwise problematic SOSIP trimers without compromising their antigenicity or structure. This new modification may have general value for HIV-1 vaccine research and development.

## INTRODUCTION

The human immunodeficiency virus-1 (HIV-1) envelope glycoprotein (Env) trimer is the sole target for virus-neutralizing antibodies (NAbs) and, therefore, is a major focus of vaccine research. HIV-1 Env, a class I fusion protein, is synthesized as a gp160 precursor protein, which is then oligomerized, extensively glycosylated, and cleaved by the host protease furin to form pre-fusion trimers composed of three gp120 and gp41 subunits (1). The function of the pre-fusion trimer is to engage with cell surface receptors (CD4 followed by CCR5 or CXCR4), and then undergo conformational rearrangements that drive virus-cell fusion (2). The trimer is necessarily metastable, and the gp120 and gp41 subunits are associated via non-covalent interactions (3).

A common approach to HIV-1 vaccine development is the use of adjuvanted, soluble Env glycoproteins. In recent years, the use of stabilized, soluble, pre-fusion trimers has become the dominant approach (4). Stabilization via protein engineering is necessary to prevent the trimer from dissociating into subunits (5). The initial, and most commonly used, design of stabilized trimers is referred to as SOSIP. These trimers contain an engineered disulfide bond between Env positions 501 and 605 that covalently links the gp120 and gp41 ectodomain subunits, and an I559P substitution in gp41 that reduces the propensity to adopt the unwanted post-fusion conformation (4). Other modifications include optimization of the furin cleavage site and truncation of the gp41 component at residue 664. The resulting SOSIP.664 construct yields stable, pre-fusion, native-like (NL) trimers, at least for some Env genotypes such as BG505 (6–9). This prototype design has since been improved in multiple ways to further enhance trimer stability and/or antigenicity (10). For example, the SOSIP.v4 design has two substitutions (64K or 66R plus 316W) that reduce exposure of CD4-induced and V3 non-NAb epitopes (11). Introducing additional intra- (73C-561C) or inter- (49C-555C) protomer disulfide bonds creates the hyper-stable SOSIP.v5 and SOSIP.v6 designs (12), while further modifications in SOSIP.v5 involve eight additional stabilizing mutations (TD8) that we have used in the SOSIP.v7 design (13, 14). The MD39 set of changes that further improve trimerization (15, 16) was also incorporated in our SOSIP.v9 design (15, 16). Finally, a repair-and-stabilize approach can also be helpful (17).

Taken together, one or more of the above protein engineering strategies usually work well enough for the production of SOSIP trimers for pre-clinical research and, increasingly, for human clinical trials. However, a subset of Env constructs is still difficult to express at adequate yields and/or purity. Some designs improve stability but reduce yield; some Env genotypes are hard to express as stabilized trimers of any design. Accordingly, there remains a need to conduct further research on SOSIP trimer stabilization to expand to an even wider range of HIV-1 Env sequences. Here, we focused on sequence changes to the gp41 ectodomain that stabilize the pre-fusion conformation.

During the virus-cell fusion process, structural rearrangements within the gp41 subunit trigger a transition from the pre-fusion to the post-fusion, six-helix bundle conformation. High-resolution structures are highly informative on these events (18–21). The gp41 ectodomain includes a fusion peptide (FP residues 512–527) and two heptad-repeat regions (HR1 residues 534–593 and HR2 residues 620–664). In the post-fusion state, HR1 and HR2 are both entirely helical and form a highly stable six-helix bundle. However, in the pre-fusion state, HR1 and HR2 exist as multiple shorter helices interspersed by unstructured regions. HR1 encompasses the α6 and α7 helices, while HR2 contributes the α8 and α9 helices (19, 20). Helix-breaking changes in HR1, such as I559P in the prototype SOSIP design, impede the transition of gp41 into the post-fusion form (6). Proline substitutions elsewhere in HR1 (22, 23), a glycine substitution (23, 24), and a redesign of the HR1 region (25) can all be effective for producing soluble, recombinant, NL trimers of the SOSIP and conceptually related designs. Moreover, the concept of using proline substitutions in HR1 for trimer stabilization has been successfully adopted and adapted for other viral vaccines based on class I fusion proteins: respiratory syncytial virus-fusion protein (RSV-F), Lassa virus glycoprotein (LASV-GP), Middle East respiratory syndrome coronavirus spike (MERS-CoV-S), severe acute respiratory syndrome coronavirus spike (SARS-CoV-S; SARS-CoV-2-S), human metapneumovirus fusion protein (hMPV-F), Ebola-glycoprotein (26).

Here, we describe how a “deletion-proline-glycine” (dPG) substitution at positions 566–568 of the HR1 region of HIV-1 gp41 increases the yield of SOSIP trimers without compromising their antigenicity or structure. We show that the dPG strategy reinforces previously described stabilization changes in existing SOSIP trimers and can rescue otherwise problematic trimers. Examples of the latter scenario include nine antigenically diverse SOSIP trimers from the global panel of HIV-1 viruses (27) that are used to quantify HIV-1 NAbs in animal and human sera (28–31). We also demonstrate the utility of the dPG change for increasing the yields of epitope knock-out (KO) BG505 SOSIP trimers used for analyzing antibody responses induced in humans and animals by the germline-targeting (GT) GT.1.1 SOSIP trimer.

## RESULTS

### Design, expression, and properties of dPG-modified BG505 Env trimers

We used existing high-resolution structures of the pre-fusion BG505 SOSIP.664 and post-fusion gp41 proteins to study the coil-to-helix transitional segments of HR1 in gp41 (18–20). Various helix-breaking proline and glycine substitutions have been incorporated into HR1 to improve trimer yield and/or quality (22–24). The helix-breaking effects differ for proline and glycine substitutions. Proline is structurally rigid, lacks an amide hydrogen for making a stabilizing hydrogen bond within an α-helix, and disrupts the helix due to the covalent attachment of its side chain to the backbone. By contrast, the lack of any side chain on glycine allows substantial backbone flexibility that underlies why this amino acid is not a preferred residue within an α-helix (32).

**Fig 1:**
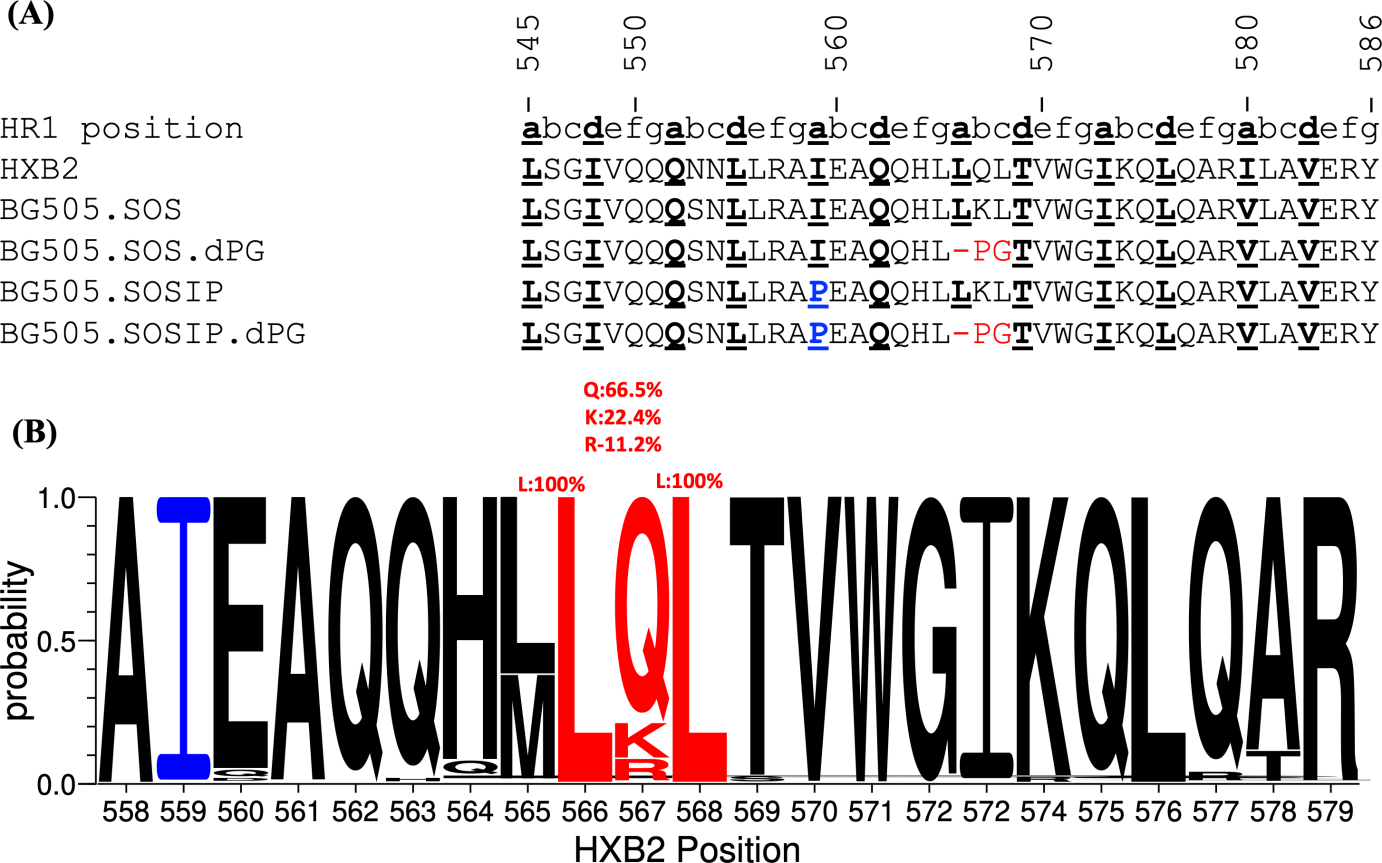
dPG modification to BG505.SOSIP.664 with or without the existing I559P change. (**A**) Amino acid sequence alignment of gp41 residues 545 and 586 of BG505 SOS (i.e., without I559P) and BG505.SOS-dPG (i.e., without I559*P* + dPG), SOSIP (i.e., with I559P) and SOSIP-dPG (i.e., with I559*P* + dPG) modified Env proteins, with changes highlighted. HR1 positions are shown as lowercase letters. HR1 repeat residues at positions “a” and “d” that correspond to the central residues of the helix coiled coil are depicted in bold. The proline amino acid substitution at I559P is highlighted in blue, and the new dPG modification in red. (**B**) A WebLogo plot, calculated using 22,128 Env sequences curated in the Los Alamos National Laboratory HIV database, is shown for positions 558-579 (HXB2 numbering). The relative height of each letter corresponds to the frequency of the amino acids at that position. Residues L566 and L568 are 100% conserved, while residue 567 is more variable (Q = 66.5%; K = 22.4%; and R = 11.2%). The conserved I559 residues are colored in blue, and dPG change positions (566–568) are colored in red.

To directly compare our new gp41 modifications to the existing I559P change, we first used the BG505 SOS.664 construct, that is, one that contains the SOS disulfide bond but not I559P, and that does not form stable trimers efficiently (33). We deleted residue 566 (d) from the gp41 subunit and substituted residues Q567 and L568 (HXB2 numbering) with proline (P) and glycine (G), respectively, to create the BG505 SOS.664-dPG construct. Deleting residue 566 at the “**a**” position disrupts the heptad repeat register (Fig. 1A). A WebLogo search shows that residues 566 and 568 are 100% conserved across the 22,128 HIV-1 Env sequences in the Los Alamos National Laboratory (LANL) HIV database (34) (Fig. 1B). Residue 567 was more variable (Q = 66.5%; K = 22.4%; and R = 11.2%), although the strong tendency for it to be a polar residue is consistent with its position in the heptad repeat.

**Fig 2:**
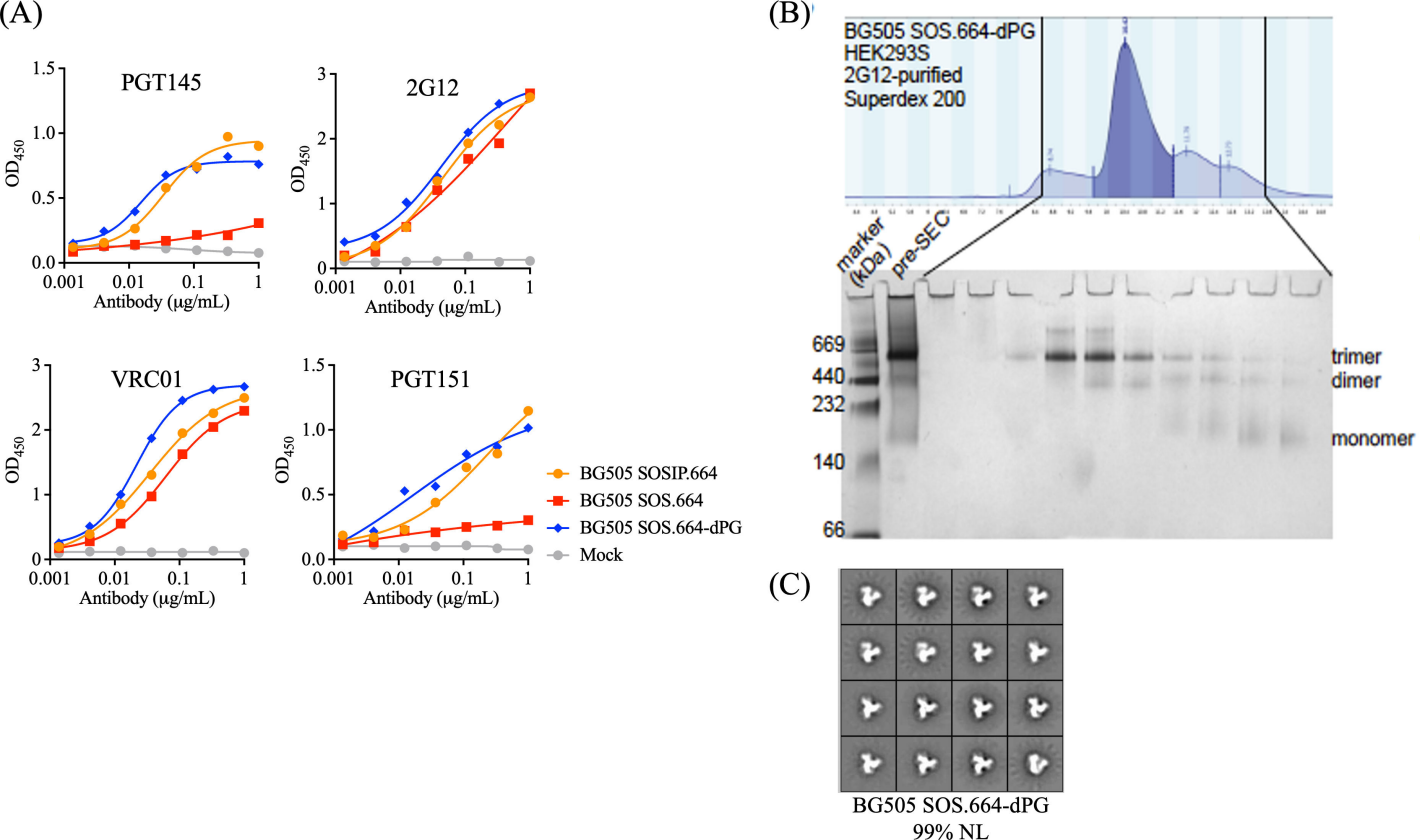
Characterization of BG505 SOS.664-dPG and related Env proteins. (**A**). D7324-capture ELISA with unpurified D7324-tagged BG505 SOS.664, SOS.664-dPG, and BG505 SOSIP.664 trimers expressed in the supernatant of HEK293T cells. (**B**) Top: SEC curve of BG505 SOS.664-dPG produced in suspension 293S cells and purified using 2G12 affinity chromatography. Bottom: BN-PAGE analysis of the SEC fractions depicted above. (**C**) NS-EM analysis on 2G12/SEC-purified BG505 SOS.664-dPG.

We expressed D7324 epitope-tagged versions of the BG505 SOS.664, SOS.664-dPG, and SOSIP.664 constructs in HEK293T cells and tested the unfractionated supernatant Env proteins using a D7324-capture ELISA (14). The goal of this initial screening experiment was to assess whether the addition of the dPG change to the SOS.664 construct would allow the production of native-like trimers, in the same way as conferred by the I559P change (i.e., as per the SOSIP.664 construct). The resulting data set confirmed that this was the case. Thus, the incompletely stabilized SOS.664 protein bound to the trimer-specific broadly neutralizing antibodies (bNAbs) PGT145 and PGT151 only very weakly, while the SOS.664-dPG protein did so comparably to the SOSIP.664 protein. Two NAbs that are not specific for native-like trimers, 2G12 and VRC01, bound all three Env proteins comparably, within an EC_50_ range of ~3-fold, confirming that each was present in the supernatants at similar levels. To verify that the SOS.664-dPG construct generated NL trimers, we used 2G12 affinity chromatography followed by size-exclusion chromatography (SEC). The SEC elution profile, analyzed by BN-PAGE, showed that trimers predominated (Fig. 2B), while negative-stain electron microscopy (NS-EM) confirmed >95% the purified trimers had a NL morphology (Fig. 2C).

**Fig 3:**
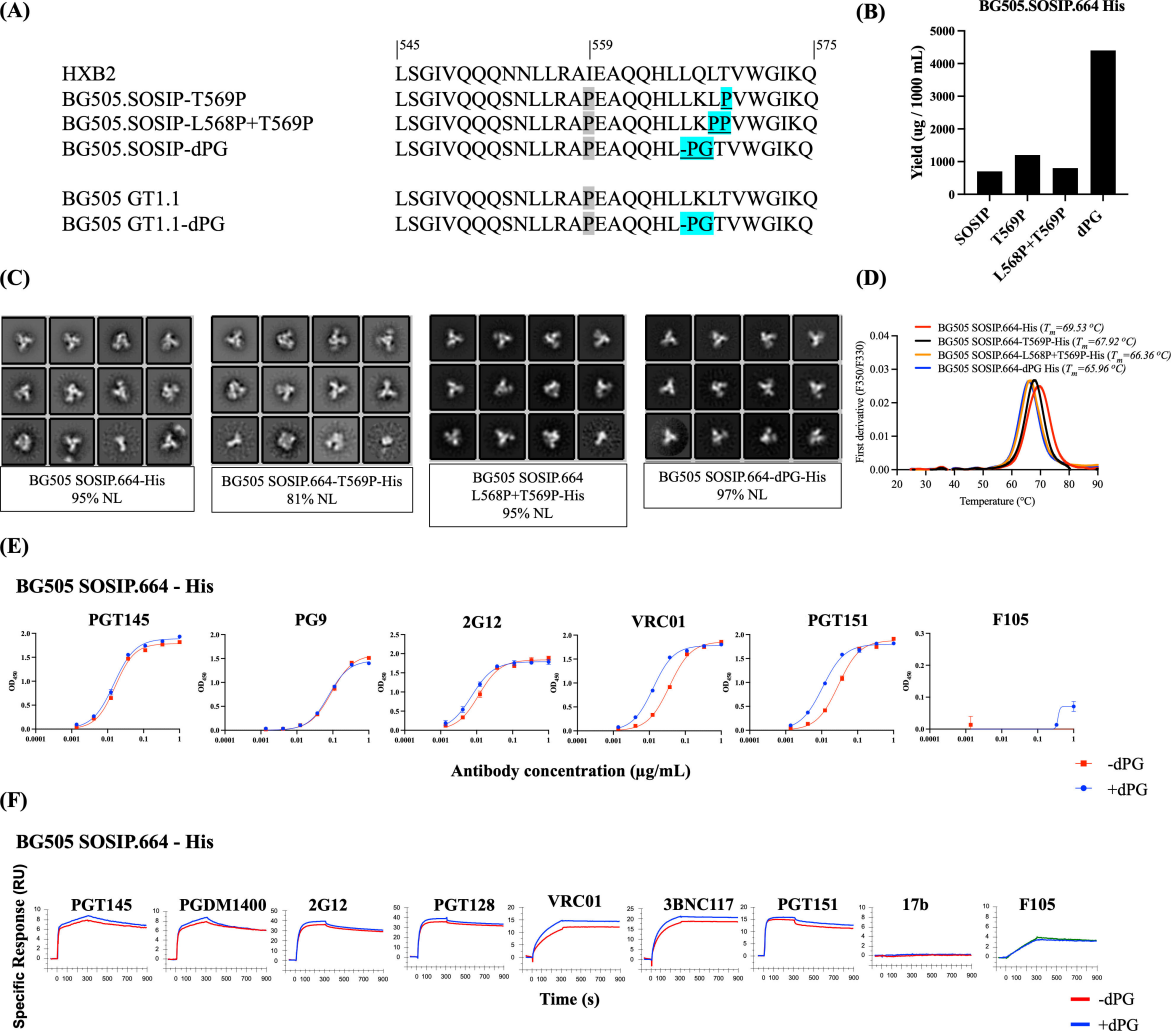
The dPG modification improves the yield, quality, and preserves bNAb epitopes of His-tagged BG505 SOSIP.664 trimers (**A**) HR1 sequence (amino acids 545–577) alignment of BG505 SOSIP.664 with its modifications (T569P, L568*P* + T569P, and dPG), and BG505 GT1.1 with or without the dPG modification. The changes are highlighted in cyan, while the I559P substitution included in all SOSIP trimers is in gray. (**B**) ExpiCHO expression yields of various PGT145 affinity-purified trimers. (**C**) NS-EM analysis on PGT145 affinity-purified BG505 SOSIP.664 and variants with the additional single proline, double proline, and dPG modifications. (**D**) DSF thermostability measurements showing the melting temperatures (T_m_) of the same BG505 SOSIP.664 and its variants. Antigenicity of dPG-modified and unmodified BG505 SOSIP.664-His. (**E**) Ni-NTA-capture ELISA binding curves for the indicated MAbs for dPG-modified and unmodified trimers. (**F**) SPR sensorgram for the indicated MAbs. The response unit (RU) difference is displayed on the y-axis as a function of time (s) on the x-axis. Each MAb (500 nM) was allowed to associate for 5 min with trimers captured at a mean level of 16 RU (with a standard deviation of 8.7 × 10^−2^ RU) on a CM3-anti-His sensor surface. MAb dissociation was recorded for 10 min. The sensorgrams depicted were double-reference subtracted and are representative of two replicate assays. Note that the y-axes have different scales that are concordant with the maximum binding extent for each MAb.

We further evaluated the impact of the dPG change on BG505 Env trimers by comparing His-tagged versions of the prototypic SOSIP.664 construct, the SOSIP.664-dPG construct, and versions containing additional single proline (T569P) (23, 35) or double proline (L568P and T569P) substitutions (22). The relevant gp41 sequence changes are shown in Fig. 3A. The various Env proteins were transiently expressed in ExpiCHO cells and affinity purified using the trimer-specific PGT145 bNAb. Compared to BG505 SOSIP.664, adding the dPG modification increased trimer yield by 6-fold (from 700 to 4,400 µg/L, respectively), whereas the additional single proline or double proline substitutions had less impact (yields of 1,200 and 800 µg/L, respectively) (Fig. 3B). NS-EM analysis showed that the PGT145-purified Env proteins expressed from each construct were predominately NL trimers (95%–97%), although the NL percentage was reduced to 81% for the T569P mutant (Fig. 3C). Differential scanning fluorimetry (DSF) revealed modest reductions (1.61°C–3.57°C) in the thermostabilities of the modified constructs compared to SOSIP.664 (T_m_ of 69.53°C) (Fig. 3D). Similar T_m_ decreases caused by dual proline (2P) substitutions have been reported elsewhere (22).

To determine whether the dPG change adversely affects the antigenicity of the BG505 SOSIP.664 trimer, we used enzyme-linked immunosorbent assay (ELISA) and surface plasmon resonance (SPR) to compare how dPG-modified and control SOSIP.664-His trimers bound to multiple neutralizing and non-neutralizing monoclonal antibodies (MAbs) against a range of epitopes. In the ELISA, the apparent strength of PGT151 and VRC01 binding was somewhat higher for the SOSIP.664-dPG trimer compared to control, with ~3-fold lower half-maximal effective concentration (EC_50_) values (Fig. 3E). Using SPR, we detected marginally greater binding by PGT145, PGDM1400, 2G12, PGT128, VRC01, 3BNC117, and PGT151 to the dPG-modified SOSIP.664 trimer (Fig. 3F). Overall, both assays show that all the bNAb epitopes present on the unmodified SOSIP trimers are preserved after dPG modification, with only modest differences in bNAb binding. By contrast, the non-NAbs F105 and 17b bound only poorly (Fig. 3E and F).

We also assessed how the dPG change affected BG505 Env pseudovirus infectivity in a single-round infectivity assay. The virus containing the dPG modification was non-infectious (Fig. S1). This outcome was expected, based on the impact of the conceptually similar helix-breaking I559P substitution (23, 36). Such modifications block conformational changes necessary for Env function.

### Cryo-EM analysis of BG505 SOSIP.664 with and without dPG modification

**Fig 4:**
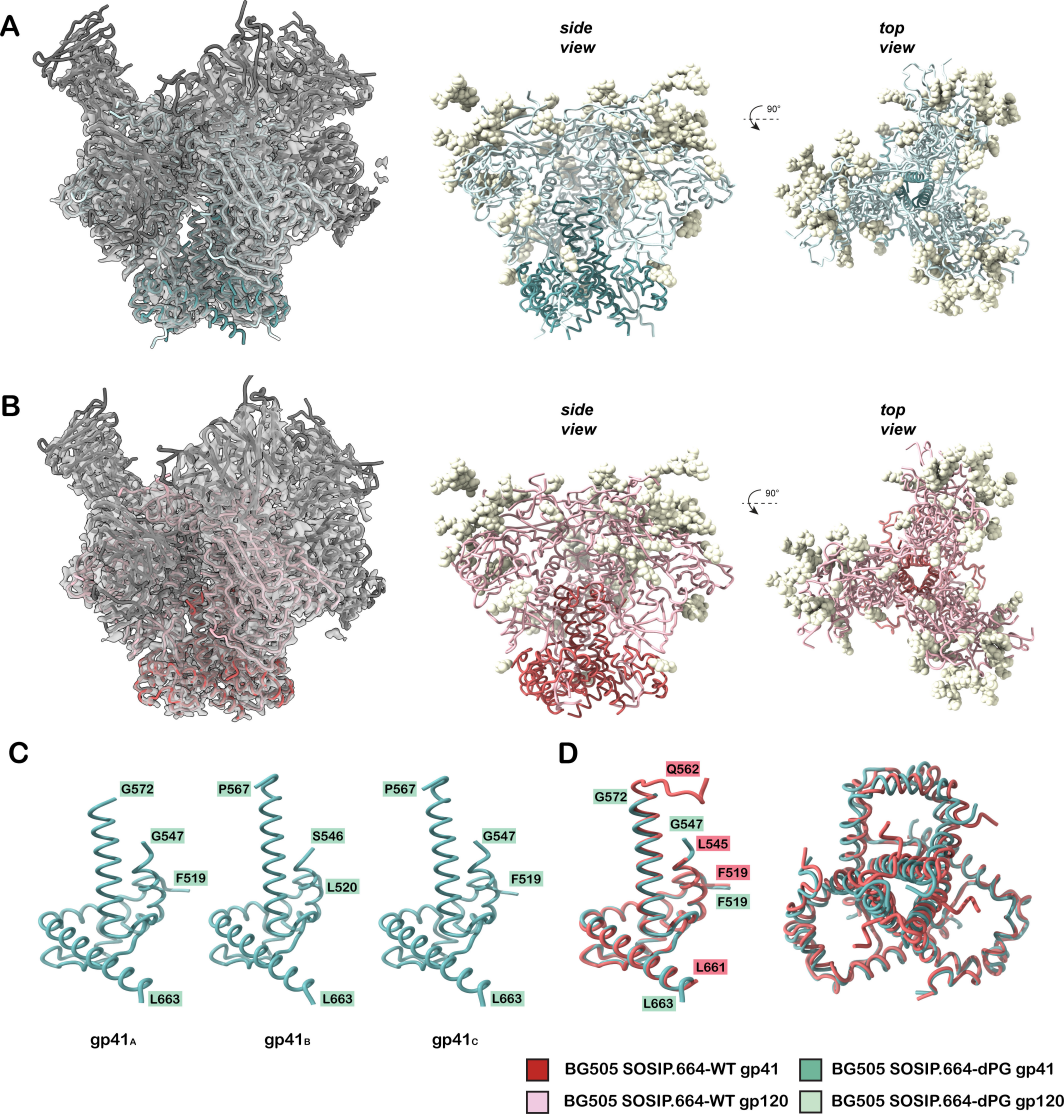
Cryo-EM analysis of BG505 SOSIP.664 with and without dPG modification. (**A**) 3.2 Å asymmetric reconstruction (transparent gray) of BG505 SOSIP.664-dPG in complex with 3BNC3117 and PGT122 Fabs with atomic model docked. (**B**) 3.0 Å C3-symmetric reconstruction (transparent gray) of BG505 SOSIP.664-WT in complex with 3BNC3117 and PGT122 Fabs with atomic model docked. In both panels A and B, the side (middle) and top (right) views of the trimer ribbon model are colored according to the key, and modeled N-linked glycans displayed as yellow spheres. (**C**) Ribbon model of each gp41 subunit of the asymmetric BG505 SOSIP.664-dPG model. Terminal residues at chain breaks are labeled according to HxB2 numbering. (**D**) Alignment of BG505 SOSIP.664-dPG gp41_A_ with BG505 SOSIP.664-WT gp41_A_ with single monomer (left) and trimer (right) displayed.

To evaluate the structural integrity of trimers with the dPG modification and probe whether it results in detectable local or global conformational changes that could affect antigenicity, we used cryogenic electron microscopy (cryo-EM). We determined high-resolution structures of BG505 SOSIP.664 and BG505 SOSIP.664-dPG in complex with bNAbs 3BNC117 (CD4-binding site [CD4bs]) and PGT122 (V3-glycan), at resolutions of 3.0 Å and 3.2 Å, respectively. The reconstructions of the trimers have similar topologies to ones previously published (18, 37) (Fig. 4A and B). As expected, each trimer was fully occupied by 3 copies of the 3BNC117 and PGT122 Fab. A close examination of the gp41 region of the wild-type trimer near the site of the dPG change showed that all three gp41 subunits were similar; the structure of this region was resolved from residue Q562 onward (note that portions of HR1 are typically disordered due to the I559P helix-destabilizing mutation). By contrast, the dPG-containing trimer had asymmetry in this region, with breaks in density prior to P567 in two gp41 subunits and prior to G572 in the third; that is, just downstream of the dPG site (Fig. 4C and D). This asymmetry and increased disorder are mostly localized to the dPG-modified region when comparing the overall gp41 trimeric topologies of the two constructs (Fig. 4D). The structures suggest that the introduction of the dPG modification further destabilizes the HR1 helix, as evidenced by increased disorder in the cryo-EM map. Nonetheless, globally, the dPG-modified construct retains the same NL conformation exemplified by SOSIP-stabilized trimers (Fig. S2E).

### The dPG modification stabilizes germline-targeting BG505 SOSIP trimers without altering their antigenicity

**Fig 5:**
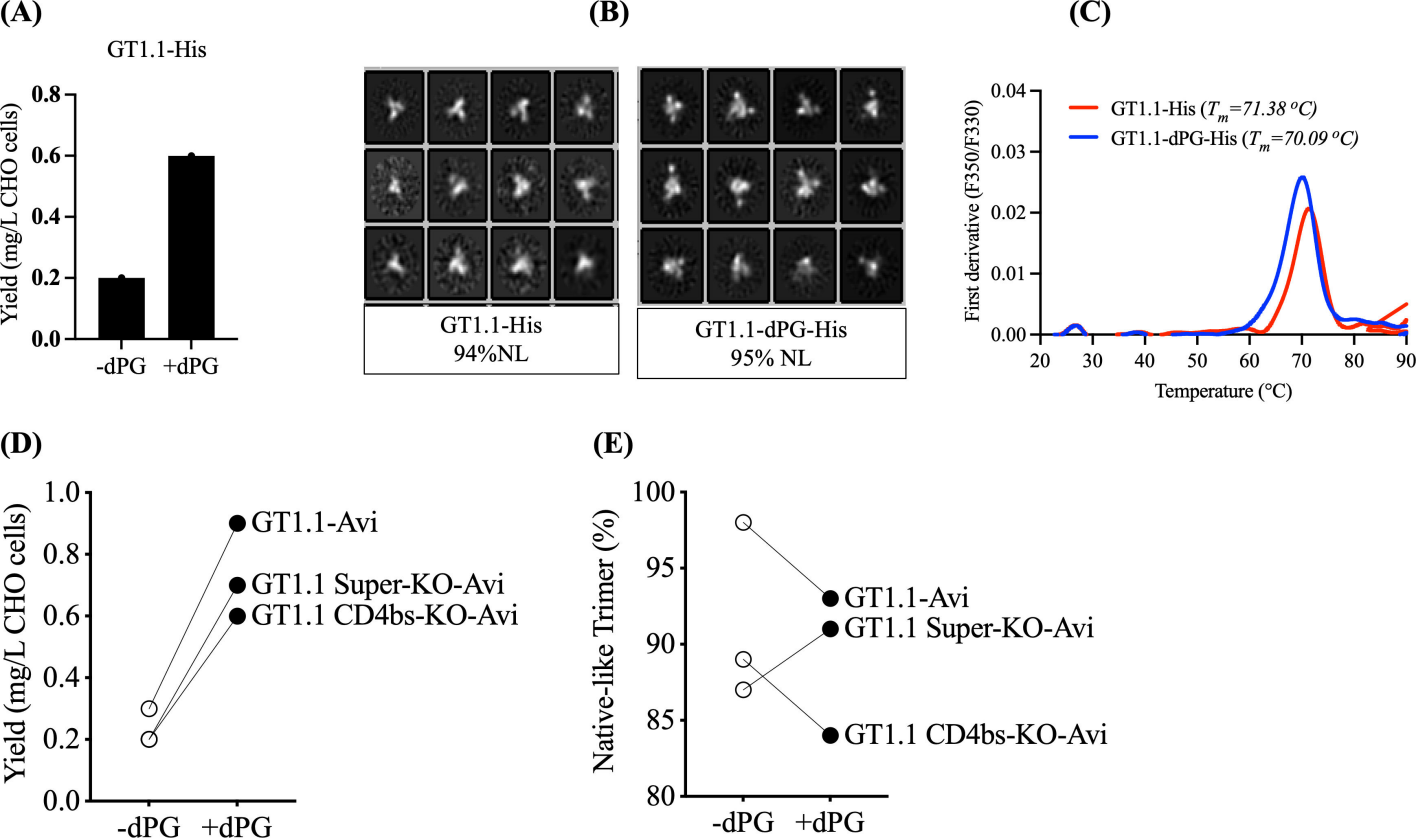
Characterization of dPG-modified and unmodified BG505 GT1.1 and epitope-KO trimer probes. (**A**) ExpiCHO expression yields of PGT145 affinity-purified His-tagged GT1.1 trimers. (**B**) NS-EM analysis on PGT145 affinity-purified His-tagged GT1.1 trimer with and without dPG modifications. (**C**) DSF thermostability measurements showing the melting temperatures (T_m_) of His-tagged GT1.1 with and without dPG modification. (**D**) The plot shows a Wilcoxon test comparison for the yield (*P* > 0.05; statistically not significant). (**E**) NS-EM analysis of analytical Avi-tagged GT1.1 and epitope-KO trimer probes with and without dPG modification.

The BG505 SOSIP.v4.1-GT1.1 trimer (from here on referred to as GT1.1) was designed to activate the pathways leading to the production of CD4bs-class (e.g., VRC01) and V2 apex bNAbs (38); it is now in clinical trials (NCT04224701, NCT05863585, NCT05983874, NCT05471076 [39, 40]). When we introduced the dPG change into the GT1.1 construct, the outcome was a threefold increase in trimer yield (from 200 to 600 µg/L) (Fig. 5A) without compromising the high NL trimer percentage of 95% (Fig. 5B). The T_m_ was only minimally altered (1.3°C) by the dPG modification (Fig. 5C). Overall, we conclude that adding the dPG change to the GT1.1 constructs increases the yield of thermostable NL trimers.

One key endpoint in the GT1.1 clinical trials is the frequency of epitope-specific (CD4bs or V2-apex) B cells present in pools of peripheral blood mononuclear cells (PBMCs) from trial participants (39, 40). For this purpose, biotin-labeled versions of the avidin (Avi)-tagged GT1.1 trimer and epitope-knockout (KO) trimer probes are required for discrimination between immunogen-induced and background signals. Examples of such analytical trimers are designated BG505 SOSIP.v8.1-GT1.1-CD4bs-KO-Avi and BG505 SOSIP.v8.1-GT1.1-CD4bs + Apex-KO-Avi. The former trimer is abbreviated to GT1.1 CD4bs-KO, while the latter is referred to as GT1.1 Super-KO (40).

Our experience has been that the highly modified epitope-KO versions of GT1.1 trimers are expressed at low yields and are relatively unstable (Fig. 5D), which complicates their production in appropriate amounts and qualities. We therefore introduced the dPG modification to the GT1.1-Avi, GT1.1 CD4bs-KO-Avi, and GT1.1 Super-KO-Avi trimer probes expressed from ExpiCHO cells. The dPG-modified GT1.1 CD4bs-KO-Avi probe was expressed at higher levels, but ~20%–30% of the resulting 2G12 plus SEC-purified trimers had non-native or open configurations (Fig. S3C). An exploratory ELISA showed that the 19b non-NAb to a V3-region epitope was reactive (Fig. S3A). This MAb is known to bind to atypically open trimers (41). We therefore used 19b as a negative selection column (42) to remove the aberrant trimers from the 2G12/SEC-purified population. Both ELISA and NS-EM confirmed the success of this procedure (Fig. S3B and C). The yield of the GT1.1 CD4bs-KO-dPG-Avi probe reported below reflects the use of the 19b negative-selection procedure. That additional purification method was not needed for the dPG-modified GT1.1-Avi and GT1.1 Super-KO-Avi probes.

Overall, dPG modification increases the yield of GT1.1-Avi (900 vs. 300 µg/L), GT1.1 CD4bs-KO-Avi (600 vs. 200 µg/L), and GT1.1 Super-KO-Avi (700 vs. 200 µg/L) trimers by ~3-fold with a NL percentage >80% (Fig. 5E). Although these probes do not consistently meet the >95% NL threshold we require for immunogens, where it is important to minimize the exposure of buried surfaces targeted by non-NAbs, our experience is that a lower threshold of >80% NL is adequate for use in the assays for which the epitope-KO trimers were designed (see below).

**Fig 6:**
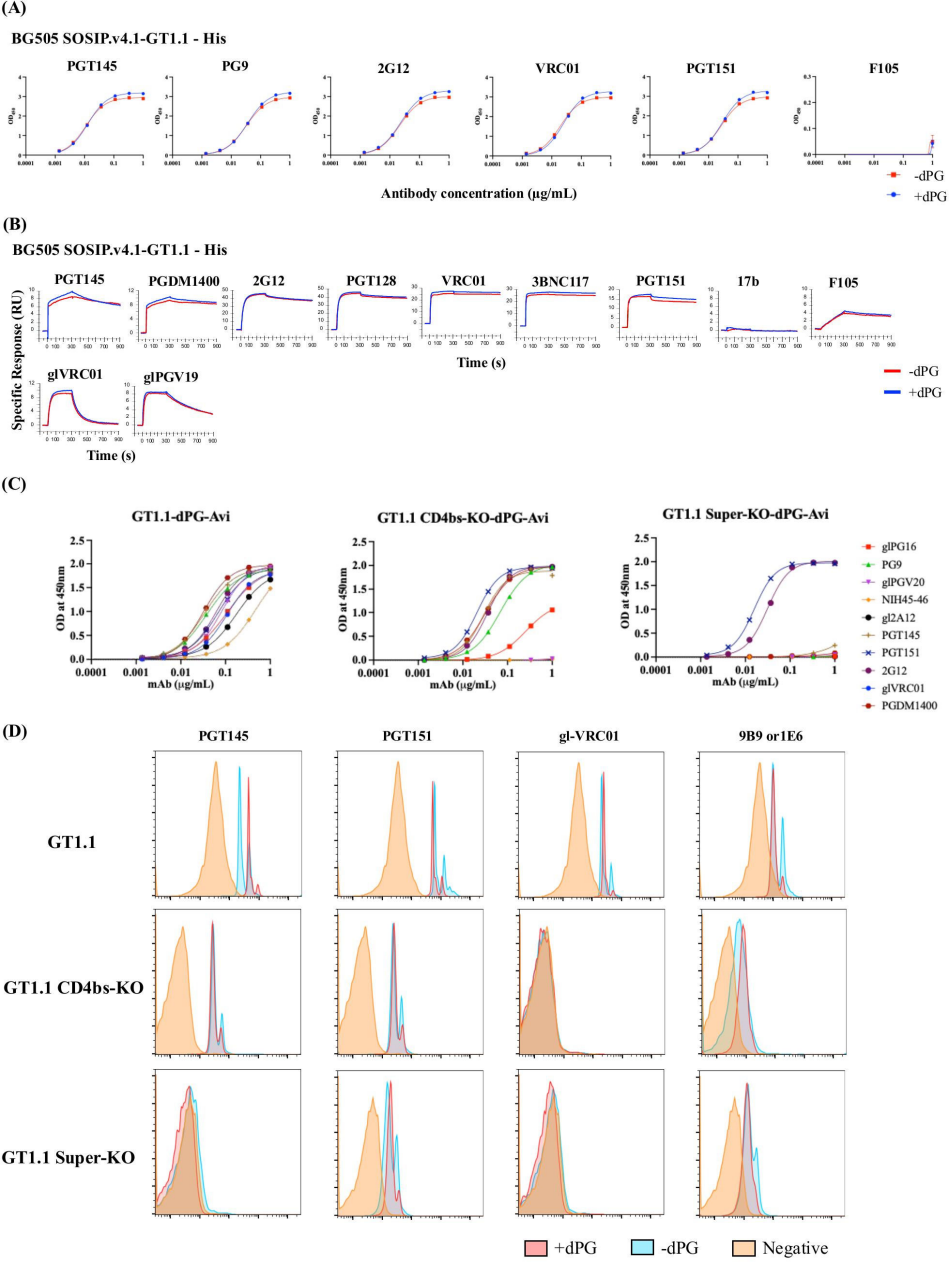
Antigenicity of dPG-modified and unmodified BG505 GT1.1 and epitope-KO trimer probes. (**A**) Ni-NTA-capture ELISA binding curves for the indicated MAbs for dPG-modified and unmodified GT1.1 trimers. (**B**) SPR sensorgram for the indicated MAbs. The response unit (RU) difference is displayed on the y-axis as a function of time (s) on the x-axis. Trimers were captured at a mean level of 17 RU (with a standard deviation of 0.19 RU) on CM3-anti-His sensor surface. Each MAb (500 nM) was allowed to associate for 5 min and dissociation was recorded for 10 min. The sensorgrams depicted were double-reference subtracted and are representative of two replicate assays. Note that the y-axes have different scales that are concordant with the maximum binding extent for each MAb. (**C**) ELISA showing antigenicity of biotinylated dPG-modified GT1.1 and epitope-KO probes. The bNAbs targeted the following epitopes: CD4bs: gl-VRC01, gl-PGV20, NIH45-46, gl-12A12; V2-apex: gl-PG16, PG9, PGT145, and PGDM1400. (**D**) Assessment of fluorochrome-conjugated GT1.1 analytical probes using bead assay: Overlay histograms of GT1.1, GT1.1 CD4bs KO, and GT1.1 Super KO analytical probes with and without dPG modification show binding to PGT145 (V2 apex), PGT151 (gp120-gp41 interface), gl-VRC01 (CD4bs), and a trimer-base non-NAb (9B9 for GT1.1 and GT1.1 CD4bs KO; 1E6 for GT1.1 Super KO). Each panel contains data for a specified trimer probe (indicated at the left side), and each histogram represents an individual test MAb (indicated at the top). The negative control (no trimer probe added) is indicated in orange. Plots are shown on a log scale.

To determine antigenicity profiles, we used ELISA and SPR to compare the GT1.1-His and GT1.1-dPG-His trimers. The binding profiles for the two GT trimer versions were similar in the two assays, while non-NAb epitopes remained minimally or non-accessible on all the trimers (Fig. 6A and B). In ELISA, all bNAbs bound to marginally higher levels against the dPG trimer; in SPR, PGT145, PGDM1400, VRC01, 3BNC117, PGT151, and glVRC01 did so. Similarly, key gl- and mature bNAb epitopes were all preserved on the biotinylated, GT1.1-dPG-Avi probe (Fig. 6C). The dPG modification also did not affect the antigenicity profiles of GT1.1 CD4bs-KO and GT1.1 Super-KO probes, although relevant CD4bs and CD4bs and Apex bNAb epitopes were knocked out, as intended (Fig. 6C).

To verify the utility of dPG-modified GT1.1, GT1.1 CD4bs-KO, and GT1.1 Super KO probes for flow cytometry, we compared their antigenicity with unmodified versions in a standardized bead assay (Fig. 6D) (40). The resulting binding profiles using PGT145, PGT151, gl-VRC01, and trimer base MAbs were similar for the two versions of each GT1.1 trimer probe. The CD4bs-KO probes did not bind to gl-VRC01, while the Super-KO probes did not react with either PGT145 or gl-VRC01, consistent with their engineered epitope-KO mutations. Hence, this quality control experiment confirms that the dPG-modified standard and epitope-KO GT1.1 probes are suitable for use in the flow cytometry assay that is part of the clinical trial analytical repertoire.

### dPG modification of SOSIP trimers based on the HIV-1 global neutralization panel

The global panel used for standardized assessment of vaccine-elicited NAb responses consists of 12 tier-2 viruses that are representative of the worldwide circulating HIV-1 strains (27). We chose to make SOSIP trimers from 9 of the 12 viruses (246F3, CE1176, CNE55, X1632, CE0217, BJOX2000, 25710, TRO11, and CH119), to complement the corresponding viruses in clinical trial analyses. For example, His-tagged or biotin-labeled Avi-tagged trimers can be used to quantify antibody binding in the Binding Antibody Multiplex Assay (BAMA) (43, 44), for antigen-specific B-cell sorting (45–47), and in studies of trimer-antibody binding kinetics using SPR or biolayer interferometry (BLI) (28, 48). Global panel trimers may also be worth evaluating as immunogens, for example, as polishing trimers in GT strategies.

**Fig 7:**
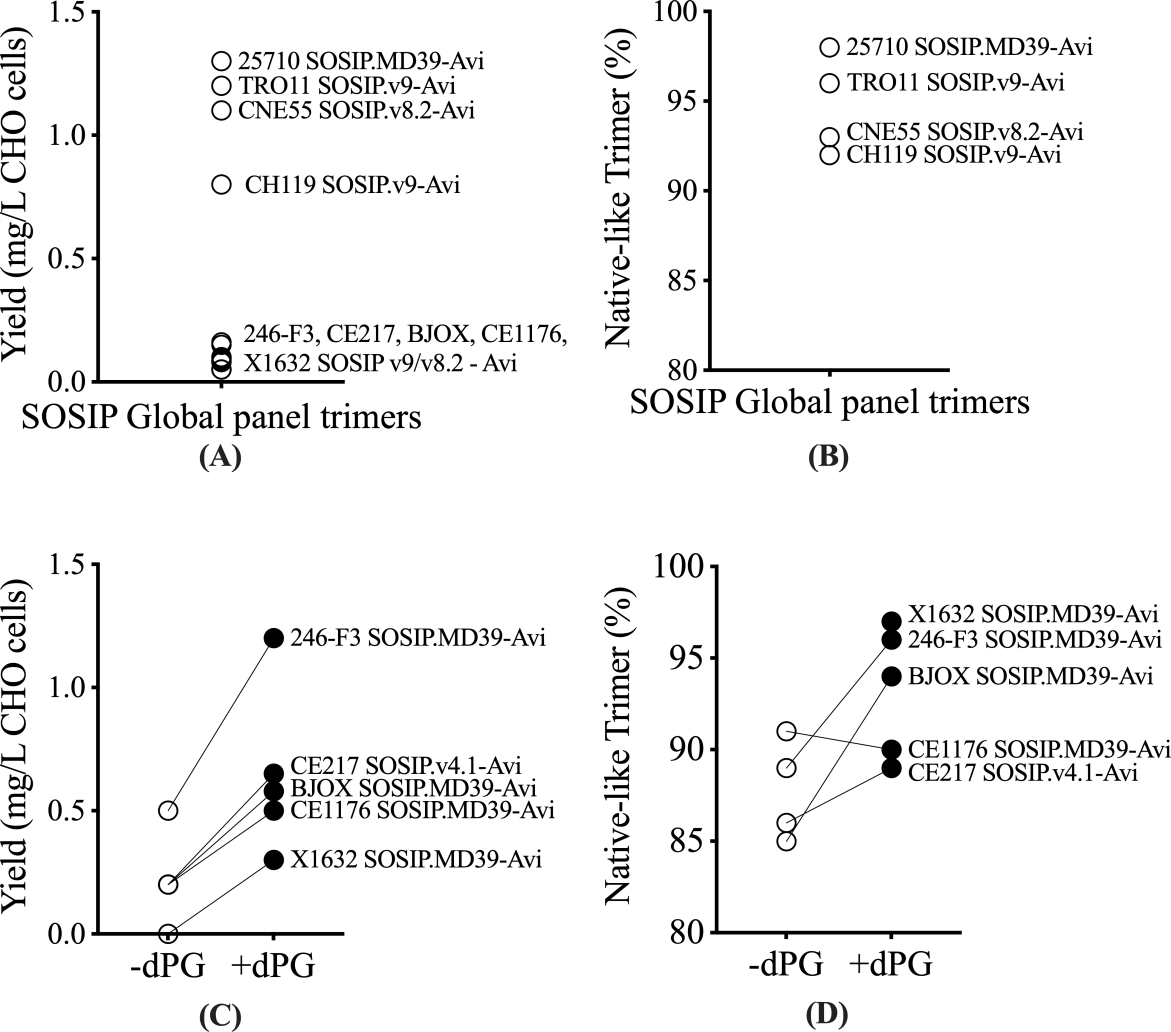
Yield and quality of HIV-1 global panel SOSIP trimers with and without dPG modification. (**A**) Yield of SOSIP trimers based on the v9, v8.2, or MD39 designs, as indicated. The affinity purification, yield, and trimer quality are summarized in Table 1. (**B**) NS-EM analysis of trimers with yields > 500 µg/L. (**C**) Wilcoxon test comparison for yields of trimers with and without dPG modification (*P* > 0.05; statistically not significant). (**D**) NS-EM analysis of trimers with and without dPG modification (not performed on X1632 without dPG due to poor expression).

To produce the nine global panel trimers, we first tested the highly stabilized v8.2 and v9 SOSIP designs (Table 1) (15, 49). When expressed transiently in ExpiCHO cells, only three of the nine constructs (TRO11 SOSIP.v9-Avi, CH119 SOSIP.v9-Avi, and CNE55 SOSIP.v8.2-Avi) produced high-quality trimers (>90% NL) in adequate yields (>500 µg/L) (Fig. 7A and B). We next assessed the SOSIP.MD39 design (16) to see whether it could improve the six problematic trimers. However, the yield of high-quality trimers was greater (1300 vs. 400 µg/L for 25710 SOSIP.v9-Avi) for only one MD39 construct, 25710 SOSIP.MD39-Avi (Table 1; Fig. 7A and B).

**TABLE 1:** Design and properties of SOSIP trimers based on the HIV-1 global panel^*a*^

Genotype	SOSIP design	Affinity purification method	Trimer yield (µg/L)	NL trimer (%)
TRO11 (Clade B)	TRO11 SOSIP.v9-Avi	PGT145	1200	96
CH119 (Clade CRF01_BC)	CH119 SOSIP.v9-Avi	2G12	800	92
CNE55 (Clade CRF01_AE)	CNE55 SOSIP.v9-Avi	PGT145	100	92
	CNE55 SOSIP.v8.2-Avi	2G12	1100	93
25710 (Clade C)	25710 SOSIP.v9-Avi	PGT145	400	94
	25710 SOSIP.v8.2-Avi	ND	ND	ND
	25710 SOSIP.MD39-Avi	2G12	1,300	98
246-F3 (Clade A1C)	246-F3 SOSIP.v9-Avi	PGT145	100	89
	246-F3 SOSIP.v8.2-Avi	PGT151	200	ND
	246-F3 SOSIP.MD39-Avi	PGT151	500	89
	246-F3 SOSIP.MD39-dPG-Avi	PGT151	1,200	96
CE217 (Clade C)	CE217 SOSIP.v9-Avi	PGT145	100	94
	CE217 SOSIP.v8.2-Avi	PGT145	100	ND
	CE217 SOSIP.MD39-Avi	2G12/SEC	200	86
	CE217 SOSIP.MD39-dPG-Avi	2G12/SEC	300	89
	CE217 SOSIP.v4.1-dPG-Avi	2G12/SEC	600	90
BJOX (Clade CRF07_BC)	BJOX SOSIP.v9-Avi	PGT145	100	93
	BJOX SOSIP.v8.2-Avi	ND	ND	ND
	BJOX SOSIP.MD39-Avi	2G12/SEC	200	85
	BJOX SOSIP.MD39-dPG-Avi	2G12/SEC	600	94
	BJOX SOSIP.v4.1-dPG-Avi	2G12/SEC	300	97
CE1176 (Clade C)	CE1176 SOSIP.v9-Avi	PGT145	100	85
	CE1176 SOSIP.v8.2-Avi	PGT151	100	ND
	CE1176 SOSIP.MD39-Avi	PGT151/SEC	200	91
	CE1176 SOSIP.MD39-dPG-Avi	PGT151/SEC	500	90
	CE1176 SOSIP.v4.1-dPG-Avi	PGT151/SEC	100	79
X1632 (Clade G)	X1632 SOSIP.v9-Avi	Poor expression		ND
	X1632 SOSIP.v8.2-Avi	Poor expression		ND
	X1632 SOSIP.MD39-Avi	Poor expression		ND
	X1632 SOSIP.MD39-dPG-Avi	2G12/SEC	300	97
	X1632 SOSIP.v4.1-dPG-Avi	2G12/SEC	200	100

^*a*^ The various SOSIP design genotype constructs from the global panel were expressed by transient transfection of ExpiCHO cells. The expressed trimers were affinity purified using the PGT145, PGT151, or 2G12, followed by SEC methods, as specified. The yield is the total amount of purified trimers produced. Trimer quality was assessed by NS-EM to estimate the NL percentage. The dPG modification was added to the five most problematic constructs (246-F3, CE217, BJOX, CE1176, and X1632) to assess its impact on yield and/or quality. ND, not done.

**Fig 8:**
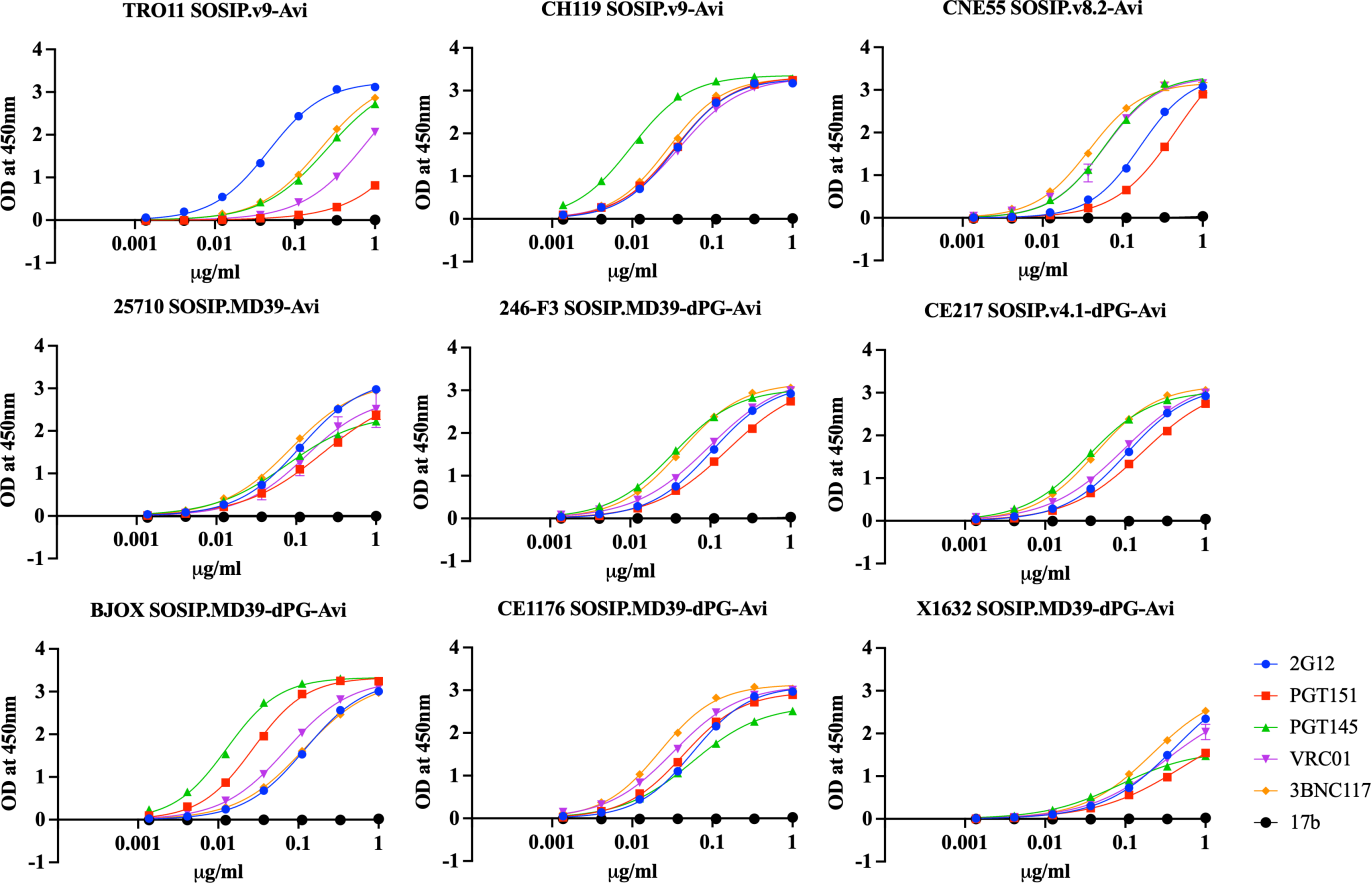
Antigenicity of HIV-1 global panel SOSIP trimers. The ELISA shows the binding of biotinylated trimers to a panel of bNAbs and non-NAbs. Four trimers could be produced at high quality and yields using an existing SOSIP or SOSIP.MD39 design (TRO11 SOSIP.v9-Avi, CH119 SOSIP.v9-Avi, CNE55 SOSIP.v8.2-Avi, 25710 SOSIP.MD39). In the remaining five cases, the dPG-modification was added to the SOSIP.MD39 or SOSIP.v4.1 design (246-F3 SOSIP.MD39-dPG-Avi, BJOX SOSIP.MD39-dPG-Avi, CE1176 SOSIP.MD39-dPG-Avi, X1632 SOSIP.MD39-dPG-Avi, and CE217 SOSIP.v4.1-dPG-Avi). Affinity purification, yield, and quality data for the global panel constructs are summarized in Table 1.

Our next step involved introducing the dPG modification into the remaining five global panel sequences (246-F3, BJOX, CE1176, X1632, and CE217). We used the SOSIP.MD39 and SOSIP.v4.1 designs as templates because these versions improved trimerization, with fewer dimers, in pilot studies. We then modified them to create the following constructs for each genotype: SOSIP.MD39-dPG-Avi and SOSIP.v4.1-dPG-Avi (Table 1). Four of the five trimers benefited from the dPG modification to the SOSIP.MD39 design (246-F3 SOSIP.MD39-dPG-Avi, BJOX SOSIP.MD39-dPG-Avi, CE1176 SOSIP.MD39-dPG-Avi, X1632 SOSIP.MD39-dPG-Avi) while the fifth was improved when the dPG changes were introduced into the SOSIP v4.1 design to create CE217 SOSIP.v4.1-dPG-Avi (Table 1; Fig. 7C and D). We assessed the antigenicity of the nine global panel trimers, five of them dPG-modified, using a MAb test panel. All the trimers bound bNAbs PGT145 and PGT151 against quaternary, trimer-specific epitopes, but none of them bound the 17b non-NAb (Fig. 8).

We compared the half-maximal effective concentration (EC_50_) values in ELISA for bNAb binding to 9 SOSIP trimers (Fig. 8) with neutralization data IC_50_ values derived using the same bNAbs and the corresponding nine wild-type Env-pseudotype viruses. The 9 SOSIP trimers used in the ELISA included the five optimally stabilized, dPG-modified versions. The neutralization data were sourced from the publicly available CATNAP database (50). The resulting comparison is presented in SI Results (Table S2). A significant Spearman’s correlation was observed for PGT151 (Spearman r, of 0.7197; *P* < 0.05), suggesting that the PGT151 epitope on Env-pseudotype viruses is well mimicked by these trimers. However, no significant correlation was observed for the other tested bNAbs (Table S2). This outcome was not unexpected given the number and nature of the stabilization changes applied to the various trimers. Nonetheless, the stabilized trimers do display all of these neutralization epitopes, which is relevant to their potential use as immunogens.

In summary, the dPG modification improved the yield and quality of the 246-F3, BJOX, CE1176, X1632, and CE217 trimer constructs that were problematic when expressed as SOSIP.v9, SOSIP.v8.2, or MD39 designs.

## DISCUSSION

The quest for an effective HIV-1 vaccine continues. The principal and well-established correlate of protection identified by active and passive immunization studies is the serum neutralization titer (51). As a result, many leading vaccine design programs now focus on immunogens intended to induce bNAbs, particularly but not exclusively SOSIP and conceptually related NL trimers (52–56). As of now, no vaccine regimen, whether trimer-based or not, has induced consistent and adequate titers of bNAbs (57, 58). A relatively new approach to the problem involves targeting gl-bNAbs by sequential immunization using priming, shaping, and polishing Env proteins delivered as adjuvanted proteins or via an mRNA platform (40, 52, 53, 59). All of these vaccination strategies would benefit from having a diverse range of SOSIP trimers as either immunogens or analytical reagents. However, the stabilities and/or yields of trimers from some HIV-1 genotypes remain problematic despite the application of various protein engineering techniques that usually but not always work well. Here, we have further modified various SOSIP trimers by introducing “deletion-proline-glycine” (dPG) changes into HR1 of gp41, a key determinant of soluble trimer stability.

The HR1 region of gp41 undergoes significant refolding during the transition from pre-fusion to the post-fusion state. The prefusion HR1 contains most of the α6-to-α7 helix, which is aligned with the C-terminal half of the post-fusion HR1 helix (19, 20). The refolding of HR1 into the post-fusion form can be impeded by stabilizing modifications, exemplified by the prototypic I559P point substitution that is an integral feature of SOSIP trimer design (4, 23, 26). Conceptually similar strategies, such as other proline substitutions, glycine substitutions, or a more extensive HR1 redesign have similarly beneficial effects on HIV-1 Env trimer stability (6, 17, 22–25), and also on the stability of glycoproteins from other viruses, including the SARS-CoV-2 Spike (26). In this study, we introduced a new HR1 modification, dPG, into various SOSIP trimers that we had found to be poorly expressed and/or unstable. This strategy deletes a highly conserved Leu at position 566 and introduces proline and glycine substitutions at positions 567 and 568, respectively. Together, these changes are predicted to disfavor the post-fusion conformation in a way that is similar to I559P and other such modifications. However, we considered it possible that the stabilization effect of dPG would be more pronounced, in particular because the dPG modification disrupts the register of the heptad repeat in addition to disfavoring helix formation. To confirm that the dPG modification did not disturb the structure of soluble trimer, we determined the cryo-EM structure of the BG505 SOSIP.664-His with and without the dPG change. The structural analysis confirmed that the dPG modification does not alter the native-like conformation of the SOSIP trimer. Similar findings were made when analyzing proline/glycine substitutions in gp41 regions that promote soluble Env trimer formation (22, 24).

Using a range of analytical methods, we found that the dPG modification increased the yield of the prototypic BG505 SOSIP.664-His and the GT BG505 GT1.1-His trimers, without adverse impact on their quality. Of particular value is the beneficial effect of the dPG change on the SOSIP.v8.1 versions of the BG505 GT1.1 CD4bs-KO and Super-KO trimers. These epitope-KO reagents are essential for analyzing B-cell responses in animal and human studies of the GT1.1 trimer (39, 47). However, the standard versions are expressed at low yields and/or quality that complicate production in the amounts projected for ongoing and planned trials. The beneficial effect of the dPG modification is therefore valuable in this context. Our development work on these and other key analytical trimers is continuing, notably by the preparation of stable CHO cell lines expressing the optimized designs (60).

Vaccine-elicited HIV-1-neutralizing antibodies are routinely assessed using the global panel of tier-2 viruses (27). SOSIP trimers based on global panel viruses could be useful as immunogens or as analytical antigens that are used in conjunction with the corresponding viruses. We therefore began a program to produce epitope-tagged versions of SOSIP trimers from a global panel that includes various clades and genotypes. As outlined in Results, some trimers from the global panel could be produced efficiently using existing SOSIP designs, but in other cases, the dPG modification was found to be beneficial to trimer yield and/or quality. We were unable to identify any “rule” as to what strategy might work best for any particular genotype; to some extent, an empirical approach needs to be taken, including an evaluation of the dPG-modification. We are now producing stable CHO cell lines (60) based on the optimal construct we have identified for each of the nine global panel trimers, in the expectation of a further improvement in yield.

Finally, we also showed that the dPG modification to full-length Env is incompatible with pseudovirus infectivity (Fig. S1). This outcome was not surprising as the change is intended to prevent the refolding to the post-fusion, 6-helix bundle that is essential for virus-cell fusion. We have not yet studied how and to what extent the dPG change might affect membrane-bound trimers, including ones expressed from mRNA platforms. However, we expect there to be benefits such as an increased expression of trimers on the cell surface. Another extension to this approach will be assessing the impact of a corresponding change in the HR1 region of class I fusion proteins of other viruses.

## MATERIALS AND METHODS

### Env protein production and purification

The HIV-1 SOSIP Env ectodomain for BG505 SOSIP.664-His and SOSIP.v4.1-GT1.1-His constructs have been described elsewhere (6, 38). They contain the gp120-gp41 disulfide bond between A501C and T605C (61); the I559P in gp41 for trimer stability (23); the REKR to RRRRRR change in gp120 for cleavage enhancement (61); a stop codon at residue 664 of gp41 to improve trimer solubility (62); and, unless otherwise specified, a C-terminal His-Tag (GSGSGGSGHHHHHHHH) for ELISA on Ni-NTA HisSorb plates and for SPR analyses. With some constructs, a C-terminal D7324 Ab epitope-tag (GSAPTKAKRRVVQREKR) was added for D7324-capture ELISA (14).

The BG505 SOSIP.v8.1-GT1.1-CD4bs-KO-Avi and SOSIP.v8.1-GT1.1-Super-KO-Avi trimers used for analyzing gl-Ab responses are derived from the GT BG505 SOSIP.v4.1-GT1.1 construct (38). Briefly, the SOSIP.v8.1 design contains all the SOSIP.v4.1 modifications (11); the A73C-A561C intra-protomeric disulfide bond (12); the TD8 substitutions (E47D, K49E, V65K, E106T, I165L, E429R, R432Q, and A500R) (13); and four MD39 substitutions (519S, 568D, 570H, and 585H) that increase thermostability and improve expression (16). The CD4bs-KO was made by introducing the N279A/D368R substitutions that ablate the binding of VRC01-class bNAbs (39). The Super-KO trimer contains the above CD4bs-KO substitutions (N279/D368R) as well as modifications to the trimer apex (N156Q, N160Q, D167G, K168E, K169V, N279A, and D368R) that eliminate the epitopes for V2 apex bNAbs (40). Different purification strategies were used that reflect our experience of what works best for each probe. Thus, the GT1.1-dPG-avi and Super-KO-dPG-Avi trimers were purified by PGT145 and PGT151 affinity purification, respectively. The GT1.1 CD4bs-KO-dPG-Avi version was purified by the 2G12/SEC method, followed by negative selection using the 19b non-NAb attached to protein A resin, as previously described (41, 42).

The dPG sequence changes (L566-deletion, Q/R567P, and L568G) were introduced using a QuickChange Site-Directed Mutagenesis kit (Agilent Technologies) and verified by sequencing (Genewiz or Plasmidsaurus). All amino acid positions are based on the HXB2 numbering system.

The HIV-1 Env global panel constructs BJOX, CE217, CE1176, CH119, CNE55, TRO11, and X1632 were expressed as the SOSIP.v8.2 and SOSIP.v9 designs (15, 16). All of these constructs contain codon-optimized genes with a C-terminal Avi-tag (GSGLNDIFEAQKIEWHE). The SOSIP.v8.2 design incorporates all of the SOSIP.v8.1 modifications described above as well as five additional MD39 substitutions (M271I, F288L, R304V, A319Y, N363Q) (16). The SOSIP.v9 construct contains all the above SOSIP.v8.2 substitutions plus an additional 49C-555C inter-protomer disulfide bond that covalently links the protomers in the trimer (15).

Global panel Env constructs that expressed poorly as SOSIP.v8.2 and SOSIP.v9 trimers were re-designed to include MD39 substitutions (16), with or without dPG modifications. Briefly, SOSIP.MD39 constructs contain the standard SOSIP.664 modifications (6) as well as 10 of the MD39 changes (T106E, M271I, F288L, R304V, A319Y, F519S, A561P, L568D, V570H, and R585H) (16). However, we excluded the N363Q substitution because it leads to an unwanted glycan hole on BG505 trimers. The dPG modifications were added to SOSIP.MD39 to create SOSIP.MD39-dPG constructs. In this design, the MD39 mutation L568D is replaced by L568G, a sequence change inherent to the dPG modification. The SOSIP.4.1-dPG construct contains SOSIP.v4.1 substitutions (38) and the dPG modifications. All the global panel constructs were codon optimized and synthesized at GeneScript (Piscataway, NJ) and then cloned into the pPPI4 expression vector.

All *env* genes were expressed by transient transfection of adherent HEK293T cells, suspension 293S or ExpiCHO cells, together with a *furin* plasmid. Briefly, HEK293T cells (ATCC, CRL11268) were maintained in Dulbecco’s modified Eagle’s medium (DMEM) supplemented with 10% fetal calf serum (FCS), penicillin (100 U/mL), and streptomycin (100 µg/mL) and transfected as described previously (11). The 293S cells (ATCC, CRL-3022) were maintained in FreeStyle medium (Life Technologies) and transfected using 1 mg/mL PEImax (Polysciences Europe GmbH, Eppelheim, Germany) at a density of 0.8-1.2 million cells/mL as described previously (14). In both cases, cell supernatants containing unpurified SOSIP trimers were harvested 3 days after transfection.

ExpiCHO cells (Thermo Fisher Scientific) were transfected as described previously (63). Briefly, on the day of transfection, the cells were diluted to a density of 6 × 10^6^ cells/mL with fresh ExpiCHO expression medium (Thermo Fisher Scientific). For 1 L of transfection, 800 µg DNA plasmids (Env +Furin; 4:1 ratio) were diluted in Opti-MEM medium (Thermo Fisher Scientific). The diluted DNA was mixed with FectoPRO transfection reagent (Polyplus, Sartorius) and used to transfect ExpiCHO cells. After 3 days, Env proteins were purified from the culture supernatant using a PGT145, PGT151, or 2G12 affinity chromatography column as previously described (6, 63, 64). The protein yield was determined using the BCA protein assay kit (Thermo Fisher Scientific).

Avi-tagged trimers were biotinylated using BirA biotin protein ligase kit (Avidity), as previously described (65).

### Env-pseudoviruses infectivity assay and western blotting

The BG505-T332N clone used for generating the Env-pseudovirus was modified to include the dPG change (BG505-T332N-dPG). The Env-pseudotyped viruses with and without dPG change were used for a single-cycle infection assay based on TZM-bl cells, as described previously (6). The native Env proteins expressed on pseudovirions were removed as described previously (66) and analyzed on the blue native polyacrylamide gel electrophoresis (BN-PAGE), followed by Western blotting as described previously (64).

### Enzyme-linked immunosorbent assay (ELISA)

For D7324-capture ELISA, supernatants from HEK293T cells containing unpurified SOSIP trimers, or PGT145-purified SOSIP trimers (1.0  µg/mL), were diluted in Tris-buffered saline (TBS). The trimers were then immobilized via their D7324 tags for 2 h at room temperature on half-well 96-well plates (Greiner) precoated with Ab D7324 (Aalto Bioreagents) at 10 µg/mL in 0.1 M NaHCO3, pH 8.6 overnight (14). The next steps are described below.

For Ni-NTA-His capture and streptavidin-capture ELISA, purified trimers containing a C-terminal His-tag or a biotinylated Avi-tag were captured onto 96-well Ni-NTA HisSorb plates (Qiagen) or Streptavidin Coated Plates (Thermo Fisher Scientific), respectively, at 200 ng/well overnight at 4°C. The unbound protein was washed away with PBS/0.05% Tween-20 (PBST) before the plate wells were blocked with 10% goat serum for 1 h. Serial dilutions for antibodies were prepared in PBS starting at 1 µg/mL, diluted by a factor of 1:3, and added to the plate (100 µL/well) and incubated at room temperature for 1 h before unbound antibody was washed away. A 100 µL aliquot of goat anti-human horseradish peroxidase (HRP)-conjugated antibody (1:3,000 dilution in PBS; Biorad) was added to the wells for 1 h. The plates were then washed and TMB substrate (Thermo Fisher Scientific) was added for 3 min. The reactions were stopped by adding 0.3N HCl. Absorbance at 450 nm was quantified using an EnSpire instrument (Perkin Elmer).

We used GraphPad Prism version 10.4.2 to analyze the ELISA data and compare trimer yields, which were also analyzed using the Wilcoxon matched-pairs test.

### Surface plasmon resonance (SPR)

The antigenicity of the standard BG505 SOSIP.664-His and the gl-targeting BG505 SOSIP.v4.1-GT1.1-His trimers, with and without dPG modifications, was determined by SPR using a BIAcore T200 instrument (Cytiva). HBS-EP^+^ (0.01 M HEPES pH 7.4, 0.15 M NaCl, 3 mM EDTA, 0.05% vol/vol Surfactant P20; Cytiva) was used as running buffer, and the experimental temperature was 25°C throughout. A panel of MAbs was selected to assess the antigenic property of His-tagged trimers after capture onto a Series S CM3 sensor chip (Cytiva) (47, 67). An anti-His antibody (His-Capture kit; Cytiva) was covalently coupled to the CM3 sensor surface via a standard amine coupling protocol with slight modifications. Briefly, the sensor surface was activated by injecting a freshly prepared mixture of EDC:NHS (1:1) for 10 min. The anti-His antibody (50 µg/mL in 10 mM sodium acetate buffer, pH 4.5) was injected for 10–15 min to achieve a density of ~4,000 response units (RU). Ethanolamine was injected for 5 min to deactivate any remaining carboxyl groups. The flow rate was kept constant at 10 µL/min during the immobilization stage. Each MAb, at a single concentration (500 nM), was allowed to associate with the captured trimer for 5 min, followed by a 10 min dissociation phase. At the end of each cycle, the sensor surface was regenerated by two 90 s pulses of glycine (10 mM; pH 1.5; Cytiva). During the binding cycle, the flow rate was 30 µL/min. The binding of each MAb was assessed individually, with two independent replicates, on a chip containing newly captured trimers. A buffer-only was conducted for each MAb under the same conditions to generate values used for the zero-analyte subtraction procedure when analyzing MAb-trimer binding data.

### Differential scanning fluorimetry (DSF)

SOSIP trimers were diluted to 0.25 mg/mL in TBS and loaded into glass capillary tubes for a thermal denaturation scan using a Prometheus NT.48 NanoDSF instrument (NanoTemper Technologies); the heating ramp was 1°C/min. The instrument software determined the thermal transition points automatically using the first derivative of the 350 nm/330 nm fluorescence ratio. First derivative melting curves were plotted using GraphPad Prism 10.

### Negative-stain electron microscopy (NS-EM)

SOSIP trimers (were diluted to ~0.03 mg/mL in TBS, adsorbed onto glow-discharged copper mesh grids, and stained with 2% (wt/vol) uranyl formate for 60 s. Automated data collection was set up using Leginon (68) on either an FEI Tecnai Spirit equipped with an FEI Eagle 4K CCD (120 kV, 2.06 Å pixel size, 52,000× magnification) or a Thermo Fisher Scientific Talos equipped with Thermo Fisher Scientific CETA 4K CMOS (200 kV, 1.98 Å pixel size, 73,000× magnification). Micrographs were saved in the Appion database (69), particles were picked using DoGpicker (70), and extraction and 2D classification were performed with Relion 3.0 (71). The 2D class averages were analyzed, and particles belonging to classes that did not unambiguously resemble NL trimers (compact, trimeric phenotypes) were marked as “non-native”.

### Cryo-EM sample preparation

Complexes of BG505 SOSIP.664 and BG505 SOSIP.664-dPG were prepared separately by incubating 150 µg of trimer and 200 µg of each monoclonal Fab (PGT122 and 3BNC117) overnight at room temperature. These two Fabs were chosen to increase angular sampling. Unbound Fab was removed by three successive washes with Tris-buffered saline (50 mM Tris pH 7.4, 150 mM NaCl) using a 100 kDa Amicon Ultra 0.5 mL centrifugal filter (Merck Millipore). The Cryo-EM grids were prepared using 3 µL of trimer-Fab complexes at a concentration of 2.6 mg/mL for BG505 SOSIP.664-dPG and 0.6 mg/mL for BG505 SOSIP.664. The trimer-Fab antigens were mixed with 0.5 µL of 0.04 mM lauryl maltose neopentyl glycol solution immediately before sample deposition onto UltrAuFoil 1.2/1.3 300 mesh grids (Electron Microscopy Services) that were glow discharged at 15 mA for 25 s. Grids were blotted for 3.5 s before being plunged into liquid ethane using a Vitrobot Mark IV (Thermo Fisher Scientific) operating at 4°C and 100% humidity.

### Cryo-EM data collection, processing, and model building

Micrographs were collected on a Glacios microscope (Thermo Fisher Scientific) operating at 200 kV equipped with a Falcon 4i detector (Thermo Fisher Scientific). Nominal magnification and pixel size were 190,000× and 0.725 Å, respectively. Automated data collection was performed using EPU (Thermo Fisher Scientific) with an approximate exposure dose of 45 e/Å2 and a nominal defocus range of −1 to −1.8 µm.

Processing was performed using CryoSPARC (72). Micrographs were curated with a CTF fit resolution upper cutoff of 10 Å. Initial particles were found using Blob Picker with a 140–240 Å diameter, particles were extracted using a box size of 560 pixels, and iterative rounds of 2D classification were performed to select trimer-Fab complexes. Following Ab initio reconstruction, particle stacks of 210,222 (BG505 SOSIP.664-dPG) and 166,433 (BG505 SOSIP.664) were subjected to Non-Uniform refinement using both C1 and C3 symmetries (73). For BG505 SOSIP.664, no structural differences were observed between the C1 and C3 maps, so the C3 map was selected. For BG505 SOSIP.664-dPG, differences were observed in the mutated (dPG) regions between the C1 and C3 maps, so the C1 map was selected. Final data collection and processing statistics are summarized (See Fig. S2; Table S1).

For model building and refinement, the BG505 SOSIP structure from PDB entry 6V0R was docked into the processed maps using UCSF ChimeraX (74) and mutated in the BG505 SOSIP.664-dPG map to match the construct used in this study. The PGT122 and 3NBC117 Fab models (PDB 4JY5 and 4JPV) were also docked into each map. Manual model building for the entire complex was performed in Coot (75, 76) and refined using real-space refinement in Phenix (77). Final models were validated using MolProbity and EMRinger in the Phenix suite, and statistics are summarized in Table S1.

### Probe preparation and flow cytometry procedures

Probes were prepared at a 1:4 molar ratio of streptavidin-fluorochrome (BV711, AX488, AX647; BioLegend) to biotinylated Avi-tagged trimer probe (GT1.1, GT1.1 CD4bs-KO or GT1.1 Super-KO with and without dPG modification). The probe, streptavidin-fluorochrome, and PBS were mixed in a microfuge tube and incubated in the dark for 60 min at 4°C. The labeled probes were stored at 4°C and used within 24 h.

Bead assays were performed to assess the functionality of probes by flow cytometry (40). Five tests were set up for each probe, four experimental and one control. Anti-mouse Ig-kappa beads (50 µL) were mixed with an equal volume of R10 media (RPMI 1640 containing 10% fetal bovine serum and 1% penicillin-streptavidin; Thermo Fisher Scientific, Waltham, MA) in polystyrene FACS tubes for each test. A 1 µg aliquot of mouse anti-human IgG (BD Bioscience, La Jolla, CA, USA) was added to each tube and incubated at 4°C for 15 min. The beads were washed with R10 and resuspended in 100 µL of the same buffer. A 1 µg aliquot of test MAb was added to the experimental but not the control tubes. These MAbs were gl-VRC01 (CD4bs), PGT145 (V2-apex), PGT151 (gp120-gp41 interface), and 9B9 or IE6 (trimer base). After incubation for 15 min at 4°C, the beads were washed and resuspended in 100 µL of R10 before the addition of the trimer probes for a further 15 min at 4°C. The beads were then washed again with R10 and resuspended in 200 µL of R10 for collection. Data from the samples were acquired on the BD S6 sorter, recorded by BD Diva software 9.5.1, and analyzed using the FlowJo 10.10 program (BD Life Sciences).

## Data Availability

The cryo-EM maps have been deposited in the Electron Microscopy Data Bank (EMDB) under accession codes EMD-70475 and EMD-70476, and the model coordinates in the Protein Data Bank (PDB) under accession codes 9OGT and 9OGU.

## SUPPLEMENTAL MATERIAL

Figures S1 to S3, and Tables S1 and S2 jvi.00913-25-s0001.docx Fig. S1: Effect of dPG modification on virus infectivity. Fig. S2: Cryo-EM data processing. Fig. S3: Negative selection with 19b non-NAb. Table S1: Cryo-EM data collection, refinement, and validation statistics. Table S2: Correlation analysis.

ASM does not own the copyrights to Supplemental Material that may be linked to, or accessed through, an article. The authors have granted ASM a non-exclusive, world-wide license to publish the Supplemental Material files. Please contact the corresponding author directly for reuse.
