# Supplementary material for: A modification to heptad repeat 1 of gp41 improves yield and/or quality of soluble pre-fusion HIV-1 envelope glycoprotein trimers
Source: J Virol. 2025 Aug 27;99(9):e00913-25. doi: 10.1128/jvi.00913-25 (PMC12455988; doi:10.1128/jvi.00913-25)
Supplement: Figures S1 to S3, and Tables S1 and S2 — Fig. S1: Effect of dPG modification on virus infectivity. Fig. S2: Cryo-EM data processing. Fig. S3: Negative selection with 19b non-NAb. Table S1: Cryo-EM data collection, refinement, and validation statistics. Table S2: Correlation analysis. [file jvi.00913-25-s0001.docx]

**Supplementary Figure S1**

**Supplementary Figure S1. Effect of dPG modification on virus infectivity.** (A) Pseudoviruses with and without the dPG modification that contained normalized amounts of p24 antigen were serially diluted and used to infect TZM-bl cells. Infectivity was measured using luciferase activity 3-days post infection. The data points shown are the means of 3 independent assays, with 3 replicates per assay. (B) The same pseudoviruses as in panel-A were solubilized and analyzed under native conditions on a Native PAGE™ 4 to 16%, Bis-Tris gel. The gel was analyzed by western blotting using a combination of the 2G12 anti-gp120, and 4E10 and RM19B1 anti-gp41antibodies.

**Supplementary Figure S2**

**
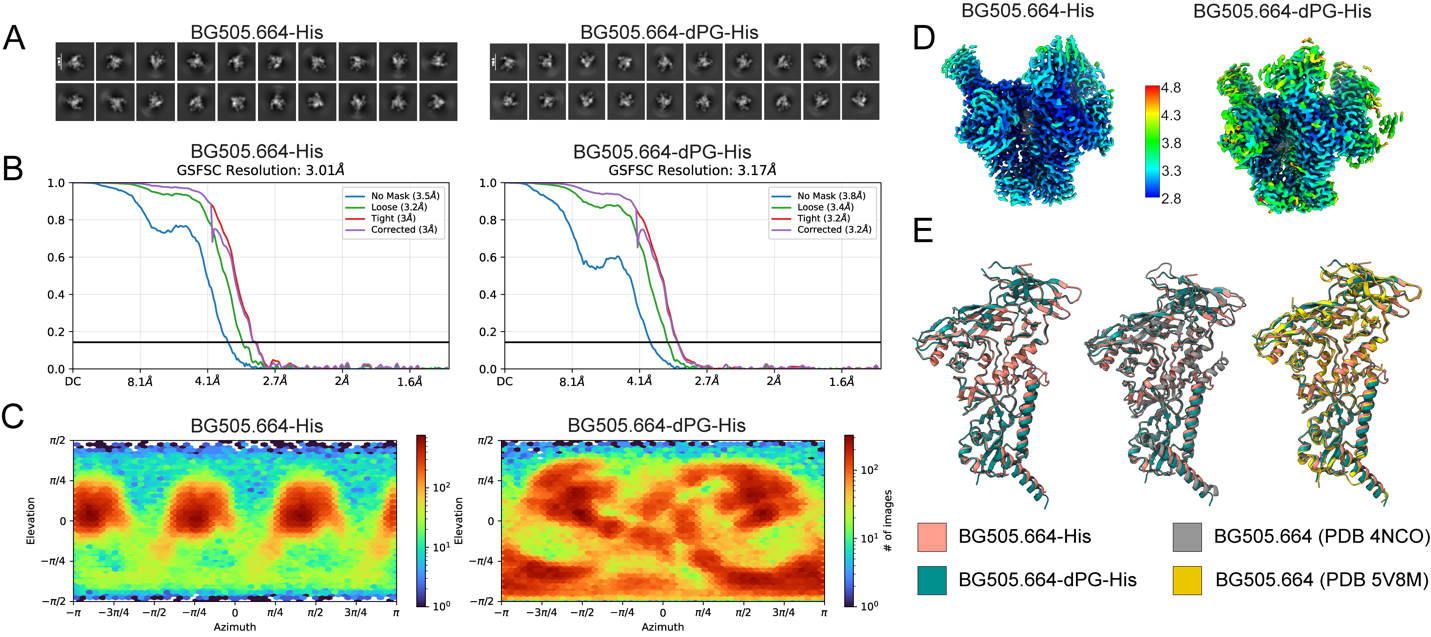
**

**Supplementary Figure S2**: **Cryo-EM Data Processing**. A) Representative 2D class averages, B) Fourier shell correlation resolution estimate, C) angular distribution of observations and D) local resolution estimation (in units Å) of BG505.664-His and BG505.664-dPG-His cryo-EM datasets. E) Superposition of cryo-EM models of BG505.664-His and BG505.664-dPG-His (gp140 protomers) with PDB 4NCO (x-ray structure of BG505 SOSIP.664 in complex with PGT122) and PDB 5V8M (cryo-EM structure of BG505 SOSIP.664 in complex with 3BNC117). Alignment based on gp120. Root-mean-square-deviation (RMSD) between BG505 SOSIP.664-His and BG505 SOSIP.664-dPG-His is 0.55 Å; between BG505 SOSIP.664-His and PDB 4NCO is 1.05 Å; and between BG505 SOSIP.664-His and PDB 5V8M is 0.84 Å.

**Supplementary Figure S3**

**Supplementary Figure S3**: **Negative selection with 19b non-NAb on GT1.1 CD4bs-KO-dPG-Avi trimers.** (A) ELISA with a panel of non-NAbs and bNAbs and the 2G12/SEC-purified biotinylated GT1.1 CD4bs-KO-dPG-Avi trimer. The non-NAbs recognize the following epitopes: F105 binds the CD4bs on open trimers; A32 binds the C1C2 region of CD4-complexed open or non-native trimers; 19b binds the V3 region of CD4-complexed open trimers; F240 binds a gp41 epitope on non-native Env proteins. (B) ELISA showing reactivity of 19b, PGT145 and 2G12 to 2G12/SEC-purified GT1.1 CD4bs-KO-dPG-Avi trimers before and after 19b negative selection. (C) NL trimer percentage of the same trimers before and after 19b negative selection as determined by NS-EM.

**Supplementary Table S1.** Cryo-EM data collection, refinement and validation statistics

|  | BG505 SOSIP.664-His + PGT122 Fab + 3BNC117 Fab  (EMD-70475)  (PDB 9OGT) | BG505 SOSIP.664-dPG-His + PGT122 Fab + 3BNC117 Fab  (EMD-70476) (PDB 9OGU) |
| --- | --- | --- |
| **Data collection and processing** |  |  |
| Microscope | TFS Glacios | TFS Glacios |
| Voltage (keV) | 200 | 200 |
| Camera | TFS Falcon 4 | TFS Falcon 4 |
| Collection mode | Counting | Counting |
| Magnification | 190,000x | 190,000x |
| Pixel size at detector (Å) | 0.725 | 0.725 |
| Total electron exposure (e–/Å^2^) | 45 | 45 |
| Exposure rate (e-/pixel/sec) | 6.17 | 6.16 |
| Number of EER frames | 40 | 40 |
| Defocus range (μm) | -1.0 to -1.8 | -1.0 to -1.8 |
| Automation software | EPU | EPU |
| Micrographs collected (no.) | 4,620 | 5,223 |
| Micrographs used (no.) | 3,982 | 4,549 |
| Initial particle images (no.) | 319,259 | 405,463 |
| Final particle images (no.) | 181,660 | 210,222 |
| Symmetry | C3 | C1 |
| Map resolution (masked/unmasked Å) | 3.0/3.5 | 3.2/3.8 |
| FSC threshold | 0.143 | 0.143 |
| Map sharpening *B* factor (Å^2^) | -97.2 | -82.8 |
| Map resolution range (Å) | 2.7-4.0 | 2.8-4.8 |
|  |  |  |
| **Refinement** |  |  |
| Initial model used (PDB code) | 5V8M, 4JY5 | 5V8M, 4JY5 |
| Refinement package | Phenix real space refine | Phenix real space refine |
| Model resolution (Å) | 3.1 | 3.3 |
| FSC threshold | 0.5 | 0.5 |
| EMRinger score | 3.45 | 2.97 |
| CC (mask) | 0.86 | 0.85 |
| *Model composition* |  |  |
| Non-hydrogen atoms | 25,401 | 25,622 |
| Protein residues | 3,030 | 3,050 |
| Ligands | 105 | 116 |
| *Mean B factors (Å^2^)* |  |  |
| Protein | 69.37 | 74.40 |
| Ligand | 74.97 | 84.38 |
| *R.m.s. deviations* |  |  |
| Bond lengths (Å) | 0.005 | 0.005 |
| Bond angles (°) | 0.906 | 1.064 |
| *Validation* |  |  |
| MolProbity score | 1.30 | 1.24 |
| Clashscore | 1.64 | 1.51 |
| Poor rotamers (%) | 0.56 | 0.83 |
| *Ramachandran plot* |  |  |
| Favored (%) | 94.39 | 95.04 |
| Allowed (%) | 5.61 | 4.96 |
| Disallowed (%) | 0.00 | 0.00 |
| Cβ outliers (%) | 0.00 | 0.00 |
| CaBLAM outliers (%) | 2.81 | 3.76 |

**Supplementary Table S2**

**Supplementary Table S2**: **Correlation between MAb binding to global panel trimers in ELISA and CATNAP neutralization values for the corresponding viruses**. Neutralization data (IC_50_, µg/ml) for the 5 MAbs tested were sourced from the CATNAP database (http://hiv.lanl.gov/catnap). The data was compared with the half-maximal effective concentration (EC_50_, µg/ml) for MAb binding to the corresponding global panel trimers, as measured by ELISA (Fig. 8). Nonparametric Spearman correlation coefficient (r), P-values, and statistical significance were analyzed using Prism software. When accurate IC_50_ values were unavailable due to a lack of neutralization, the highest concentration tested was included in the correlation analysis (i.e., if the IC_50_ value was recorded as >50 μg/ml, we used a value of 50 μg/ml).
